# Unexpected Ground-Glass Opacities on Abdominopelvic CT of a Patient With a Negative SARS-CoV-2 Antigen Test Result and No Respiratory Symptoms Upon Admission

**DOI:** 10.7759/cureus.11044

**Published:** 2020-10-19

**Authors:** Carol Soler-Luna, Domingo Reynoso-Saldana, Monica I Burgos, Cesar H Gutierrez

**Affiliations:** 1 Internal Medicine, University of Texas Rio Grande Valley School of Medicine/Doctors Hospital at Renaissance, Edinburg, USA; 2 Anesthesiology, Universidad Autonoma de Guadalajara, Guadalajara, MEX

**Keywords:** abdominopelvic computed tomography, admission, antigen tests, asymptomatic, covid-19, ground-glass opacities, negative predictive value, sars-cov-2

## Abstract

One of the biggest challenges during the coronavirus disease 2019 (COVID-19) pandemic continues to be the detection of asymptomatic and presymptomatic persons infected with severe acute respiratory syndrome coronavirus 2 (SARS-CoV-2). Persons infected with SARS-CoV-2 who do not have symptoms of COVID-19 may transmit the virus to others and may have subclinical lung abnormalities. Some hospitals use SARS-CoV-2 antigen tests for pre-admission screening testing because they are relatively inexpensive, have a rapid turnaround time, and can be performed at the point of care; however, antigen tests are generally less sensitive than nucleic acid amplification tests with reverse transcription polymerase chain reaction (RT-PCR) assay. Moreover, as the local COVID-19 prevalence increases, the negative predictive value of antigen tests may decrease, meaning that the probability of having false-negative results may increase. We present a case of a patient who, prior to admission for a surgical procedure, had a negative antigen test result for SARS-CoV-2, had no respiratory symptoms, and had no suspected or known exposure to SARS-CoV-2; however, she tested positive for SARS-CoV-2 RNA after admission. The only factor that led the healthcare team to suspect SARS-CoV-2 infection was an unexpected finding of bilateral ground-glass opacities on an abdominopelvic computed tomography (CT), which was performed to assess the extent of a perianal abscess the patient presented. This case highlights the importance of using highly sensitive SARS-CoV-2 tests for pre-admission screening testing in the hospital setting.

## Introduction

The severe acute respiratory syndrome coronavirus 2 (SARS-CoV-2), formerly known as 2019-nCoV, causes the coronavirus disease 2019 (COVID-19), which at the end of 2019 started as a cluster of pneumonia cases of unknown etiology in Wuhan, Hubei province, central China [1-3]. SARS-CoV-2 binds the angiotensin-converting enzyme 2 (ACE2) host receptor and uses the host transmembrane protease serine 2 (TMPRSS2) for cell entry [1,4]. Symptoms of COVID-19 include fever, cough, shortness of breath, myalgia, headache, sore throat, diarrhea, nausea and vomiting, loss of smell or taste, and rhinorrhea [5]. A common pattern on chest computed tomography (CT) of symptomatic patients is ground-glass opacity [6]. Nevertheless, there have been reports of people infected with SARS-CoV-2 who did not develop symptoms of COVID-19, transmitted the virus to others, and had incidental CT findings of ground-glass opacities [7]. Furthermore, many institutions conduct screening testing using nucleic acid amplification testing (NAAT) with reverse-transcription polymerase chain reaction (RT-PCR) assay for SARS-CoV-2 upon a patient’s admission to the hospital, but other institutions conduct screening testing with antigen tests for SARS-CoV-2, which are generally less sensitive than NAATs [8]. Here we report a case of a patient who did not present with respiratory tract symptoms, was not suspected of having COVID-19, and tested negative for a SARS-CoV-2 antigen test upon admission for a surgical procedure; however, an abdominopelvic CT scan incidentally revealed bilateral ground-glass opacities consistent with viral pneumonia.

## Case presentation

A 61-year-old woman was admitted to the hospital with fever, leukocytosis, and severe pain in the anal area. The patient had been in her usual state of health until seven days before this admission, when perianal pain developed. She was evaluated by her primary care physician, who found an erythematous area in her perianal skin and prescribed her a course of oral clindamycin and an antibiotic cream. Seven days later, she returned to her primary care physician because her pain worsened. Ketorolac and empiric ceftriaxone were administered, and she was sent to the emergency department.

In the emergency department, the patient presented with severe perianal pain. She had a history of diabetes mellitus, hypertension, obesity, hypercholesterolemia, and hypothyroidism. Her medications included telmisartan, dapagliflozin, exenatide, atorvastatin, and levothyroxine. Shortness of breath, cough, smell or taste disturbances, and myalgia were not reported. No suspected or known exposure to SARS-CoV-2 was reported. On examination, the temperature was 100.1°F, blood pressure 110/52 mmHg, heart rate was 85 beats per minute, respiratory rate was 19 breaths per minute, and the oxygen saturation was 100% while the patient was breathing ambient air. The lungs were clear on auscultation. In the perianal area, there was an abscess that had begun to drain spontaneously. The white cell count was 11,400/µL (reference range: 4,800-10,900/µL), lactic acid was 2.32 mmol/L (reference range: 0.5-1.99 mmol/L), and anion gap was 14 mEq/L (reference range: 2-12 mEq/L). A decision for admission of the patient was made; therefore, a SARS antigen fluorescent immunoassay (FIA) screening test was performed, and the result was negative. Morphine and acetaminophen were administered for pain and fever. The surgery department was consulted, and a combination of vancomycin with piperacillin-tazobactam was recommended. In addition, a CT of the abdomen and pelvis was performed after the administration of intravenous contrast material.

**Figure 1:**
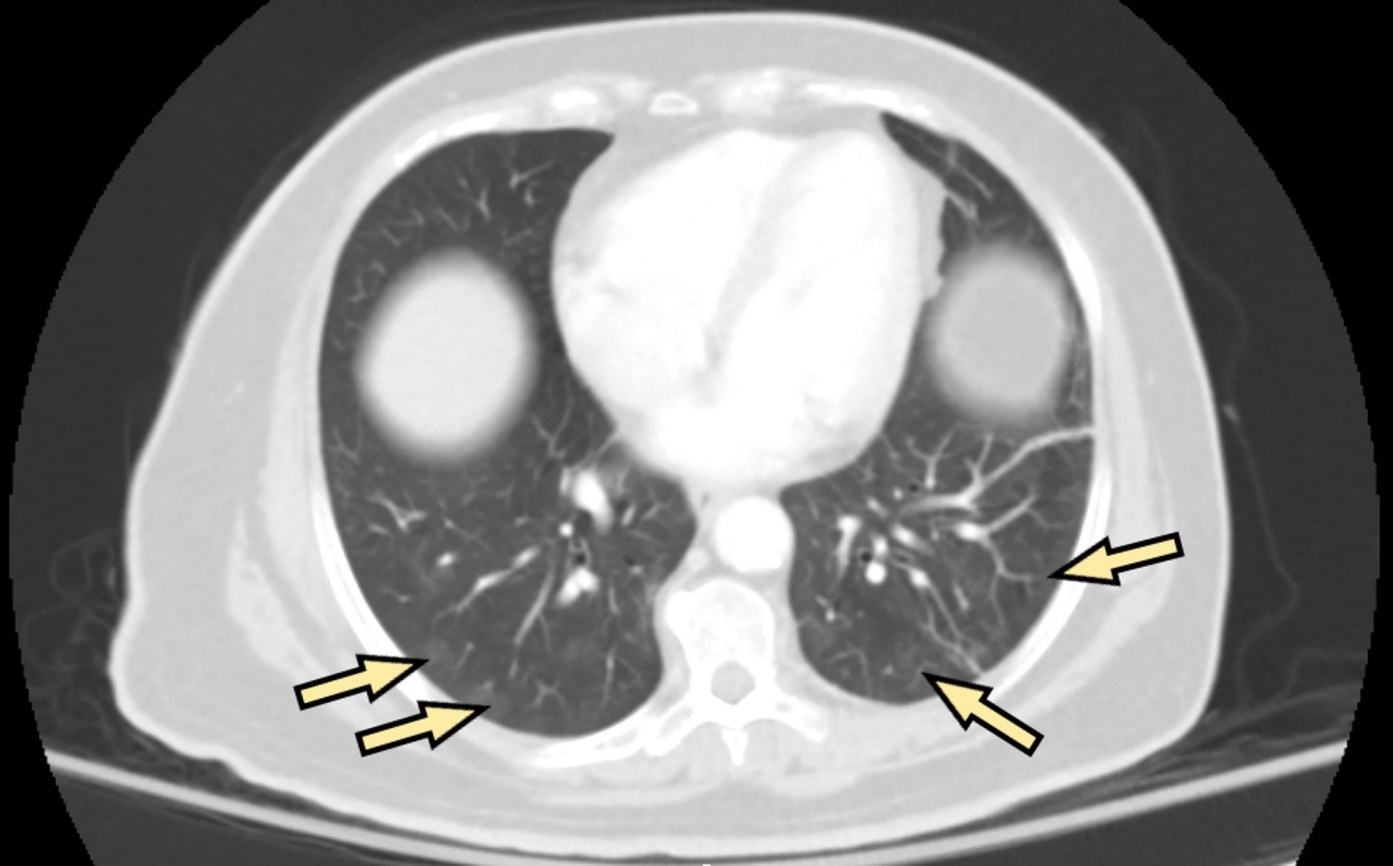
Bilateral peripheral ground-glass opacities in the lung bases (arrows) of a 61-year-old woman, unexpectedly noted on axial abdominopelvic CT performed to assess the extent of a perianal abscess.

The abdominopelvic CT scan revealed a loculated fluid collection posterior to the anus consistent with an abscess. In addition, there were peripheral ground-glass opacities in both imaged lung bases (Figure 1). Five hours after the initial presentation, the patient was admitted. A nasopharyngeal swab was obtained for NAAT. Six hours after admission, the test was positive for SARS-CoV-2 RNA and the patient was transferred to the COVID-19 unit. A posteroanterior radiograph of the chest was performed, and the radiologist reported slightly increased interstitial markings, especially in the lung bases. Ten hours later, incision and drainage of the patient’s abscess were successfully performed in the operating room. The postoperative diagnoses included horseshoe perirectal abscess, perianal abscess, and asymptomatic SARS-CoV-2 infection. No specific therapy for COVID-19 was administered to the patient because she had no respiratory symptoms.

Three days after admission, a chest radiograph was performed, which did not reveal any abnormalities. The next day, the patient continued without any respiratory symptoms, and the perianal pain had significantly decreased. The vital signs were normal. The white cell count, the value of the serum anion gap, and the serum lactate levels were normal. The patient was subsequently discharged home with a prescription of levofloxacin. During her follow-up visit with the surgery department 36 days later, she did not report any symptoms whatsoever.

## Discussion

Early identification of asymptomatic (i.e., infected persons who will not develop symptoms) and presymptomatic (i.e., infected persons who will eventually develop symptoms) SARS-CoV-2 infections continues to be a challenge, even more so when these patients do not have known or suspected exposure to SARS-CoV-2. In a report released by the United States Centers for Disease Control and Prevention (CDC), among 373,883 laboratory-confirmed COVID-19 cases with data on individual symptoms, 70% reported fever, cough, or shortness of breath, 36% reported myalgia, and 34% reported headache; in addition, sore throat, diarrhea, nausea and vomiting, loss of smell or taste, and rhinorrhea were significantly reported [5]. In one study of 1,099 patients with laboratory-confirmed COVID-19 from Wuhan and other areas in China, ground-glass opacity was the most common radiologic finding on chest CT of symptomatic patients upon admission [6]. On the other hand, the proportion of infections with SARS-CoV-2 that are asymptomatic and presymptomatic is not known. A narrative review analyzed three cohorts with representative samples and estimated the asymptomatic infection rate as high as 40% to 45%, with a conservative estimate of 30% or higher to account for the presymptomatic infections that were not quantified; however, prospective studies with large and representative samples of the general population need to be conducted so as to better reflect the population at large [7]. Although our patient presented with fever in the emergency department, which is a common symptom of COVID-19, we can assume that she was a case of asymptomatic SARS-CoV-2 infection because all her symptoms were very consistent with her abscesses.

Pre-admission screening testing for SARS-CoV-2 is intended to identify infected persons who are asymptomatic or presymptomatic without known or suspected exposure to SARS-CoV-2 so that infection control interventions can be taken to prevent further transmission [9]. For pre-admission screening testing, NAAT with RT-PCR to detect SARS-CoV-2 RNA is preferred over antigen testing (i.e., tests that detect SARS-CoV-2 antigen) because it is generally more sensitive (i.e., it has fewer false-negative results); however, some hospitals use antigen tests because they are relatively inexpensive, have a faster time to results compared to some NAATs, and can be performed at the point of care [8]. Furthermore, there are limited data to guide the use of rapid antigen tests as screening tests in asymptomatic and presymptomatic persons to detect or exclude SARS-CoV-2 infection [8]. Our patient tested negative for a SARS antigen FIA test as part of the pre-admission screening policy of the hospital, which was a false-negative result because she tested positive for an RT-PCR test thereafter, with a time interval between collection of samples for the two tests of about 8 hours as per CDC guidelines.

As of September 18, 2020, the U.S. Food and Drug Administration (FDA) has granted emergency use authorizations (EUAs) to four SARS-CoV-2 antigen tests [10]. Antigen levels in specimens collected beyond five to seven days of the onset of symptoms may drop below the limit of detection of these tests [8]. The negative predictive value (NPV) of these tests is the probability that a patient who has a negative test result truly does not have SARS-CoV-2 infection [8]. The NPV may vary depending on the test sensitivity and the pretest probability (i.e., an estimate, before testing, of the person’s chance of being infected), which, in turn, might depend on local COVID-19 prevalence, SARS-CoV-2 exposure history, and symptoms [11]. For instance, assume that the local COVID-19 prevalence equals the pretest probability and the test specificity is 100%; if the local COVID-19 prevalence were 20% and the sensitivity 95%, the NPV would be 99% (1% of negative test results would be false negative); however, with a prevalence of 50% and a sensitivity of 70%, the NPV would be 77% (23% of negative test results would be false negative) [11]. This means that as the local prevalence of COVID-19 increases and tests with lower sensitivity than NAATs (e.g., antigen tests) are used, the probability of having false-negative results may increase even with a test specificity of 100%, which highlights the importance of using highly sensitive SARS-CoV-2 tests for pre-admission screening.

Several studies have reported ground-glass opacities as one of the most common CT findings in asymptomatic persons infected with SARS-CoV-2 [12,13]. Nonetheless, data on CT findings in asymptomatic persons infected with SARS-CoV-2 are limited. One important limitation is the lack of longitudinal data collection over a sufficiently long time to distinguish between asymptomatic and presymptomatic cases [7]. An abdominopelvic CT was performed on our patient to assess the extent of her perianal abscess, which unexpectedly showed bilateral ground-glass opacities in the lung bases, consistent with viral pneumonia.

The fact that an incidental abdominopelvic CT scan finding of bilateral ground-glass opacities was the only factor that prompted our team to suspect SARS-CoV-2 infection demonstrates how challenging it is to detect asymptomatic and presymptomatic infections in the healthcare setting and how it is possible to have SARS-CoV-2 asymptomatic infections with subclinical lung abnormalities. When pre-admission or pre-procedure screening tests fail to detect asymptomatic or presymptomatic patients in the hospital setting, it creates a false sense of security to the patient and the healthcare team caring for the patient, prevents infected patients from being isolated or housed with other similarly infected patients, and increases the risk of infection among vulnerable patients.

## Conclusions

The amount of testing for SARS-CoV-2 must be increased for patients without symptoms of COVID-19 in the general population, even without known or suspected exposure to SARS-CoV-2, due to the likely high proportion of asymptomatic and presymptomatic infected persons transmitting the virus to others. Furthermore, asymptomatic and presymptomatic persons may have subclinical lung abnormalities, as detected by CT as ground-glass opacities. On the other hand, the healthcare team should perform pre-admission screening testing for SARS-CoV-2 with tests that have higher sensitivity than antigen tests (e.g., NAAT with RT-PCR) and short turnaround time at the point of care. If resources do not allow these practices and antigen tests are used, a negative antigen test result should be considered presumptive based on its lower NPV in the context of high local prevalence of COVID-19; this may also be considered even in places with low reported prevalence due to the unknown proportion of asymptomatic infections.

## References

[1] Zhou P, Yang XL, Wang XG (2020). A pneumonia outbreak associated with a new coronavirus of probable bat origin. Nature.

[2] Gorbalenya AE, Baker SC, Baric RS (2020). The species severe acute respiratory syndrome-related coronavirus: classifying 2019-nCoV and naming it SARS-CoV-2. Nat Microbiol.

[3] (2020). Novel Coronavirus (2019-nCoV) Situation Report - 1. https://www.who.int/docs/default-source/coronaviruse/situation-reports/20200121-sitrep-1-2019-ncov.pdf.

[4] Hoffmann M, Kleine-Weber H, Schroeder S (2020). SARS-CoV-2 cell entry depends on ACE2 and TMPRSS2 and is blocked by a clinically proven protease inhibitor. Cell.

[5] Stokes EK, Zambrano LD, Anderson KN (2020). Coronavirus disease 2019 case surveillance — United States, January 22-May 30, 2020. MMWR Morb Mortal Wkly Rep.

[6] Guan W, Ni Z, Hu Y (2020). Clinical characteristics of coronavirus disease 2019 in China. N Engl J Med.

[7] Oran DP, Topol EJ (2020). Prevalence of asymptomatic SARS-CoV-2 infection. Ann Intern Med.

[8] (2020). Interim Guidance for Rapid Antigen Testing for SARS-CoV-2. https://www.cdc.gov/coronavirus/2019-ncov/lab/resources/antigen-tests-guidelines.html.

[9] (2020). Interim Infection Prevention and Control Recommendations for Healthcare Personnel During the Coronavirus Disease 2019 (COVID-19) Pandemic. https://www.cdc.gov/coronavirus/2019-ncov/hcp/infection-control-recommendations.html.

[10] (2020). In Vitro Diagnostics EUAs. https://www.fda.gov/medical-devices/coronavirus-disease-2019-covid-19-emergency-use-authorizations-medical-devices/vitro-diagnostics-euas.

[11] Woloshin S, Patel N, Kesselheim AS (2020). False negative tests for SARS-CoV-2 infection — challenges and implications. N Engl J Med.

[12] Hu Z, Song C, Xu C (2020). Clinical characteristics of 24 asymptomatic infections with COVID-19 screened among close contacts in Nanjing, China. Sci China Life Sci.

[13] Inui S, Fujikawa A, Jitsu M (2020). Chest CT findings in cases from the cruise ship “diamond princess” with coronavirus disease 2019 (COVID-19). Radiol Cardiothorac Imaging.

